# Supplementary material for: Genomic Analysis of Gastrointestinal Parasite Resistance in Akkaraman Sheep
Source: Genes (Basel). 2022 Nov 22;13(12):2177. doi: 10.3390/genes13122177 (PMC9778220; doi:10.3390/genes13122177)
Supplement: Supplementary file 1 [file genes-13-02177-s001.zip › genes-2014911-supplementary.pdf]

Supplementary Table S1. The number of infected and non-infected animals.

Traits	Infected (N)	Non-infected (N)	Non-infected (%)
**NemEPG3**	43	86	68.59
**MonEPG3**	81	48	38.01
**CocOPG3**	60	69	4.95
**NemEPG6**	29	446	93.90
**MonEPG6**	149	326	68.63
**CocOPG6**	298	177	5.68
